# RNA sequencing-based transcriptome analysis of granulosa cells from follicular fluid: Genes involved in embryo quality during in vitro fertilization and embryo transfer

**DOI:** 10.1371/journal.pone.0280495

**Published:** 2023-03-01

**Authors:** Eun Jeong Yu, Won Yun Choi, Mi Seon Park, Jin Hee Eum, Dong Ryul Lee, Woo Sik Lee, Sang Woo Lyu, Sook Young Yoon

**Affiliations:** 1 CHA fertility Center Gangnam, CHA University, Seoul, Korea; 2 CHA Fertility Center Seoul Station, CHA University, Seoul, Korea; 3 CHA Fertility Center Ilsan Woman’s & Children’s General Hospital, CHA University, Gyeonggi-do, Korea; 4 Department of Biomedical Science, CHA University, Gyeonggi-do, Korea; South China Agricultural University, CHINA

## Abstract

**Background:**

Granulosa cells play an important role in folliculogenesis, however, the role of RNA transcripts of granulosa cells in assessing embryo quality remains unclear. Therefore, we aims to investigate that RNA transcripts of granulosa cells be used to assess the probability of the embryonic developmental capacity.

**Methods:**

This prospective cohort study was attempted to figure out the probability of the embryonic developmental capacity using RNA sequencing of granulosa cells. Granulosa cells were collected from 48 samples in good-quality embryo group and 79 in only poor- quality embryo group from women undergoing in vitro fertilization and embryo transfer treatment. Three samples from each group were used for RNA sequencing.

**Results:**

226 differentially expressed genes (DEGs) were related to high developmental competence of embryos. Gene Ontology enrichment analysis indicated that these DEGs were primarily involved in biological processes, molecular functions, and cellular components. Additionally, pathway analysis revealed that these DEGs were enriched in 13 Kyoto Encyclopedia of Genes and Genomes pathways. Reverse transcription quantitative polymerase chain reaction verified the differential expression of the 13 selected DEGs. Among them,10 genes were differently expressed in the poor-quality embryo group compared to good-quality embryo group, including CSF1R, CTSH, SERPINA1, CYP27A1, ITGB2, IL1β, TNF, TAB1, BCL2A1, and CCL4.

**Conclusions:**

RNA sequencing data provide the support or confute granulosa expressed genes as non-invasive biomarkers for identifying the embryonic developmental capacity.

## Introduction

The ability to identify oocyte quality remains one of the most significant challenges in assisted reproductive technology. The quality of oocyte is an important predictor of implantation and live birth [1, 2]. The presence of a mature and high-quality oocyte plays an essential role in the development of a high-quality embryo [3]. This means that the selection of high-quality embryos begins at the time of oocyte selection.

Embryo quality is a strong indicator for the success rate of in vitro fertilization (IVF) program, as the live birth rate increases when good quality embryos are transferred [4]. The incidence of only poor-quality embryos is usual in IVF cycles of patients with advanced maternal age and in low responding patients. According to Semondade et al. [5], the incidence rate of only poor embryos is approximately 10% at the first IVF cycle, and the recurrence rate is 3%.

Morphological features and development rates are key indicators for oocyte and embryo selection during IVF. These methods are familiar for both clinician and embryologist and are used as the standard method in embryos selection step. However, the usefulness of their assessment is being questioned because of personal bias from the embryologist [6]. In recent years, techniques for embryo selection that provide chromosomal analysis to improve clinical pregnancy have recently been developed, such as preimplantation genetic testing (PGT) [7]. Although PGT is a strong predictor for implantation, it is expensive and requires invasive embryo biopsy, which involves technical expertise [8]. Therefore, there is a high demand for a non-invasive and easy-to-perform screening tools to improve the selection of the most pregnancy-competent embryo.

Good-quality and mature oocyte in IVF are important for fertilization and embryo development [9]. Oocyte maturation occurred during folliculogenesis through orchestrated cross-talk between the oocyte and granulosa cells (GCs). Therefore, it is advocated that the functions of GCs indirectly reflect oocyte developmental competence. GCs can be easily recovered in large quantities during oocyte collection. Thus, the gene expression analysis in GCs could provide a non-invasive assessment for identifying the most competent oocytes and embryos. However, the transcriptomic analysis of GC used to identify embryo quality is still controversial. While some studies have identified candidate genes expressed in GC that could be expected as biomarkers of oocyte and embryo quality [10, 11] and successful clinical outcomes [12–14], others have reported that there are no significant differences in gene expression between embryos that did or did not successfully implant [15, 16].

Therefore, the present study aims to investigate and compare the GC transcriptomic obtained from subjects producing good-quality embryos and those producing only poor quality embryos in human IVF. We carried out RNA sequencing (RNA-seq) of GCs isolated from follicular fluid to identify novel gene transcription factors correlated with embryo quality among the embryos with good quality.

## Materials and methods

### Study population

This prospective cohort study was conducted between January 2019 and February 2021 at the CHA Fertility Center Gangnam. The study was approved by the CHA University Gangnam CHA hospital institutional review board (GCI-19-10, May 15, 2019), Republic of Korea. All women gave written informed consent to provide material for this study. All procedures followed the rules for studies with human-origin materials established by the IRB. Of 141 embryos transfer (ETs), 48 were good quality (GQ) and 79 were poor quality (PQ) ETs; 14 cases of GQ and PQ ETs cases with endometriosis, endometrial pathologies, uterine fibroids or hydrosalpinx were excluded. Couples with severe male factor infertility as defined based on severe oligozoospermia (<5 million sperm/mL) or a history of testicular biopsy were excluded.

### Ovarian stimulation and embryo transfer procedures

Patients underwent controlled ovarian stimulation using either the midluteal long gonadotropin-releasing hormone (GnRH) agonist protocol or GnRH antagonist protocol. Gonadotropin doses were individualized according to patients’ age, anti-Mullerian hormone level, antral follicle count, and previous response to stimulation. Cycle monitoring with transvaginal ultrasonography and serum estradiol measurement, was continued until hCG administration. When at least two follicles reached 18 mm in diameter, 250–500 μg of recombinant hCG was administered for final oocyte maturation. Transvaginal ultrasound-guided oocyte-retrieval under conscious sedation was carried out 34–36 hours after hCG administration. The mature oocytes were inseminated using intracytoplasmic sperm insemination (ICSI). One or two embryos were transferred to each patient, and ETs occurred on day 3, 4, or 5. All patients received luteal phase support with progesterone after ET until 8–10 weeks after pregnancy.

### Isolation of GCs and assessment of embryo quality

Follicular fluids were aspirated and pooled for each patient during oocyte-retrieval. Follicle aspirates, which were not clear and were contaminated with endometriosis cysts, were discarded. Granulosa cells recovered from one woman were used as one sample. The 127 samples were divided into two groups according to the patient’s embryo quality; 48 patients with good quality blastocysts having a grade of at least 3BB and 97 patients with only poor quality blastocysts.

The follicular fluid was centrifuged at 1000 x g for 20 min to separate erythrocytes, leukocytes and GCs. The cell pellets were washed in RBC lysis buffer (Roche Diagnostics, Basel, Switzerland) for RBC removal and centrifuged at 500g for 10 min and separated enzymatically with instigation at 37°C for 30 min in the enzyme solution [Hanks’ balanced salt solution (HBSS, Gibco, Grand Island, NY) containing 0.5 mg/ml collagenase (type IV; Gibco) and 0.25mg/ml dispase II (neutral protease, grade III, Roche)]. The suspension of GCs was washed and filtered through a 40μm mesh (BD, Franklin Lakes, NJ). GCs using TRIzol Reagent (Life Technologies, San Diego, CA, USA) were stored -75°C until analysis.

Embryo grading was evaluated by two embryologists and was assessed by another senior embryologist with over at least ten years of work experience before embryo transfer. For standardization, all embryologists at our center were trained in embryo grading by the same laboratory director. Embryo quality assessment was carried out according to previously described protocol [17]. Briefly, good quality embryos were identified using the following characteristics. Cleavage embryos were classified as GQ according to Cummins criteria [18, 19]. Blastocyst quality was assessed regarding to the degree of blastocoel expansion, inner cell mass (ICM) and trophectoderm (TE) morphology on day 5 or 6 [20].

### RNA isolation and NGS library preparation

Total RNA was isolated from cells using TRIzol Reagent (Invitrogen, Carlsbad, CA, USA) according to the manufacturer’s instructions. RNA integrity was checked by using an Agilent 2100 BioAnalyzer (Agilent, CA, USA) with an RNA integrity number value greater than 6. Only qualified samples were used for RNA library constitution. The libraries were arranged for 151 bp paired-end sequencing by the TruSeq stranded mRNA sample preparation kit (Illumina, CA, USA). After the sequential process of end repair, A-tailing, and adapter ligation, cDNA libraries were amplified by Polymerase Chain Reaction (PCR). They were quantified using the KAPA library quantification kit (Kapa Biosystems, MA, USA) following the manufacturer’s quantification protocol. After cluster amplification of denatured templates, sequencing was carried out as paired-end (2×151bp) using IlluminaNovaSeq6000 (Illumina, CA, USA).

### Transcriptome data analysis

#### DEG analysis

Based on the estimated read counts in the previous step, DEGs were screened using the R package TCCv.1.26.0 [21]. The TCC package uses well-set normalization strategies to compare the tagcount data. Normalization factors were estimated by the iterative DESeq2 [22] /edgeR method [23]. The Q-value was assessed based on the p-value using the p.adjust function of the R package by default parameter programs. The DEGs were evaluated with a Q- value threshold of < 0.05 for fixing errors due to multiple testing.

#### GO analysis

The GO database indicates a set of hierarchically controlled vocabulary classified into three categories: biological process, cellular component, and molecular function. For functional characterization of the DEGs, GO-based trend test was assessed by the R package called GOseq [24] through the Wallenius non-central hypergeometric distribution. Selected genes with P-values < 0.05 following the test were regarded as statistically significant.

### RT-qPCR

Approximately 1 μg RNA from each sample was reverse transcribed to cDNA using a SensiFAST™ cDNA Synthesis Kit (Meridian Bioscience, Tennessee, USA) following the manufacturer’s instructions. Thirteen genes were selected for validation from the list of DEGs. Primers were designed using the Primer3 primer-design program (version 0.4.0; http://bioinfo.ut.ee/primer3-0.4.0/) (S1 Table). The qPCR reactions were performed on the CFX Connect Real-Time PCR detection System (Bio-Rad Laboratories, Inc., USA) using iQ™ SYBR® Green Supermix (Bio-Rad Laboratories, Inc., USA). Relative quantification analyses were carried out using the comparative CT method, and relative gene expression levels were calculated using the 2^−ΔΔCT^ method.

### Clinical outcome and statistics

The primary outcomes of the study were clinical pregnancy outcomes, including clinical pregnancy rate, and ongoing pregnancy rate. Clinical pregnancy was defined as the presence of at least one gestational sac with a fetal heartbeat on ultrasonography. Ongoing pregnancy was defined when a positive heartbeat was at 12 weeks or more of gestation on ultrasonography. All statistical analyses were conducted using SPSS (version 25.0; Chicago, IL, USA) software. Categorical variables were performed using the chi‐square and Fisher’s exact tests. Continuous variables were assessed using Student’s *t* test. A probability (*p*) value <0.05 was considered to determine statistical significance. A *p* value below 0.05 was considered statistically significant.

## Results

### Study population

**Fig 1:**
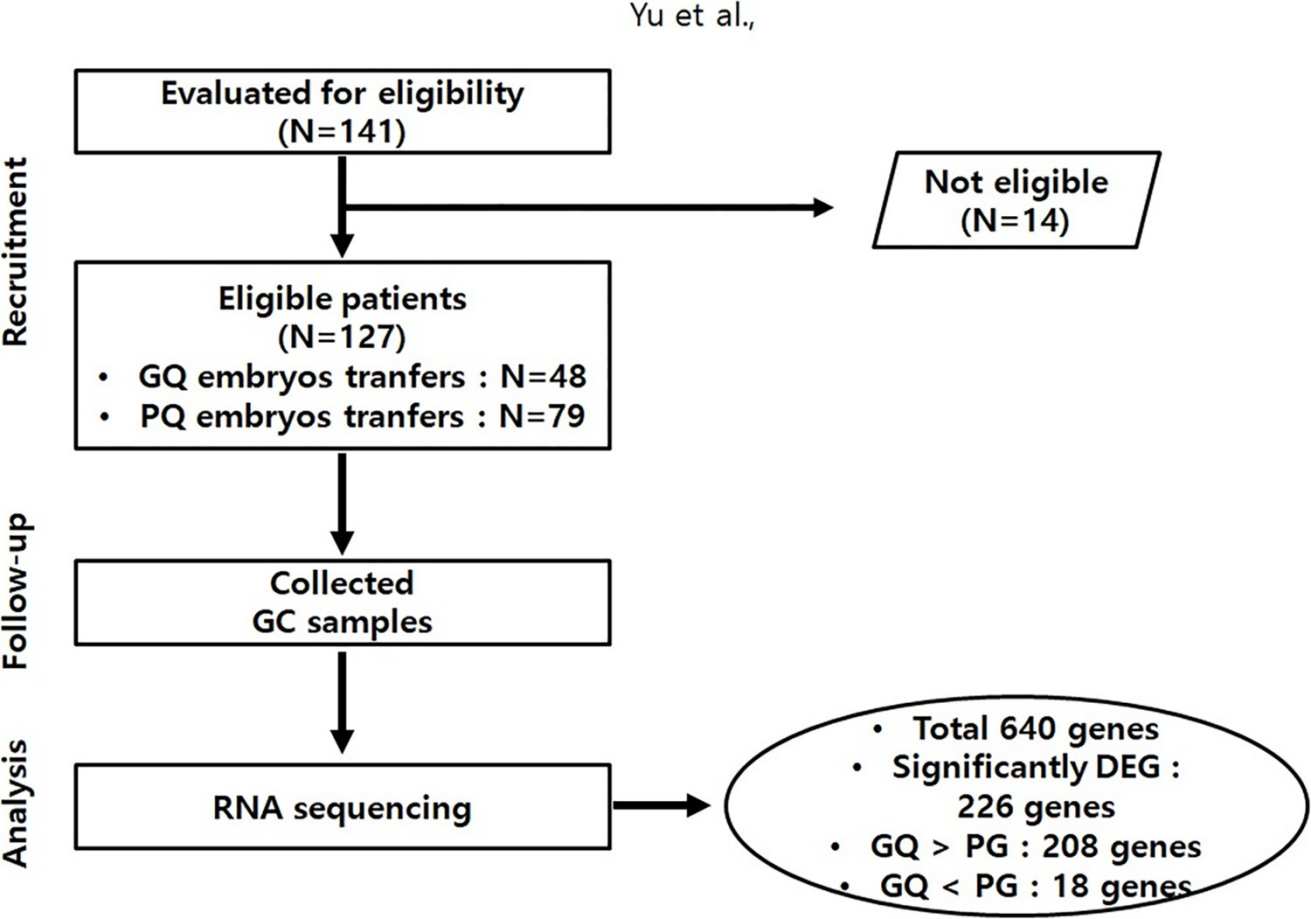
Study flowchart.

There were 141 women undergoing IVF and embryo transfers (ETs), which included 48 women who had good-quality (GQ) ETs, 79 with only poor-quality (PQ) ETs, and 14 with either GQ or PQ that were excluded from this study (Fig 1). Most patients (n = 83) underwent double ETs, 59 samples came from a single ET, and 3 from triple ETs. ETs for 91 samples occurred on day 3 or 4, and for 50 on day 5, no significant difference between the GQ and PQ groups according to the day of ET.

Table 1 presented the characteristics of patients between the GQ and the PQ groups. There were no significant differences in age, body mass index, infertility duration, and anti-Mullerian hormone level. However, there were more ETs in the PQ group than in the GQ group. The clinical pregnancy rate was higher in the GQ (52.0%) than in the PQ embryo transfer group (40.5%); however, the difference was statistically insignificant (*p*  =  0.09), and the ongoing pregnancy rate of the GQ group was significantly higher than that of the PQ group (45.8% vs. 24%, respectively, *p*  =  0.02).

**Table 1:** Patient characteristics for the analysis of granulosa cell gene expression associated with embryo quality.

	Good-quality(GQ) embryo (N = 48)	Poor-quality(PQ) embryo (N = 79)	*P*-value
**Age (yr)**	35.3 ± 2.9	36.3 ± 3.7	0.11
BMI (kg/m^2^)	20.3 ± 4.2	21.5 ± 3.2	0.48
**Infertility duration (yr)**	3.6 ± 2.3	3.4 ± 2.5	0.54
**Previous IVF attempts (n)**	1.4 ± 0.8	2.2 ± 1.8	0.001
**AMH (ng/mL)**	3.2 ± 2.7	2.7 ± 2.4	0.26
**Basal E2 (pg/mL)**	40.2 ± 17.8	54.3 ± 41.1	0.02
**Basal FSH (mIU/mL)**	7.4 ± 2.7	7.9 ± 2.6	0.17
**Days of stimulation**	12.7 ± 1.3	12.8 ± 1.3	0.51
**E2 on hCG day (pg/mL)**	2376.3 ± 1301.1	1872.2 ± 926.0	0.01
**LH on hCG day (mIU/mL)**	4.4 ± 3.4	4.1 ± 2.6	0.57
**P4 on hCG day (ng/mL)**	0.6 ± 0.4	0.5 ± 0.4	0.24
**Number of retrieved oocytes**	13.0 ± 6.9	10.7 ± 4.9	0.04
**Number of transferred embryos**	1.5 ± 0.7	1.9 ± 0.4	0.01
**Clinical pregnancy rate**	54.1% (26/48)	31.6% (25/79)	0.02
**Ongoing pregnancy rate**	50% (24/48)	26.5% (21/79)	0.04

BMI, body mass index; IVF, in vitro fertilization; AMH, anti-Mullerian hormone; E2, estradiol; FSH, follicle-stimulating hormone; LH, luteinizing hormone; P4, Progesteron 4; hCG, human chorionic gonadotropin

### Identification of differentially expressed genes (DEGs): samples clustered according to embryo quality

**Fig 2:**
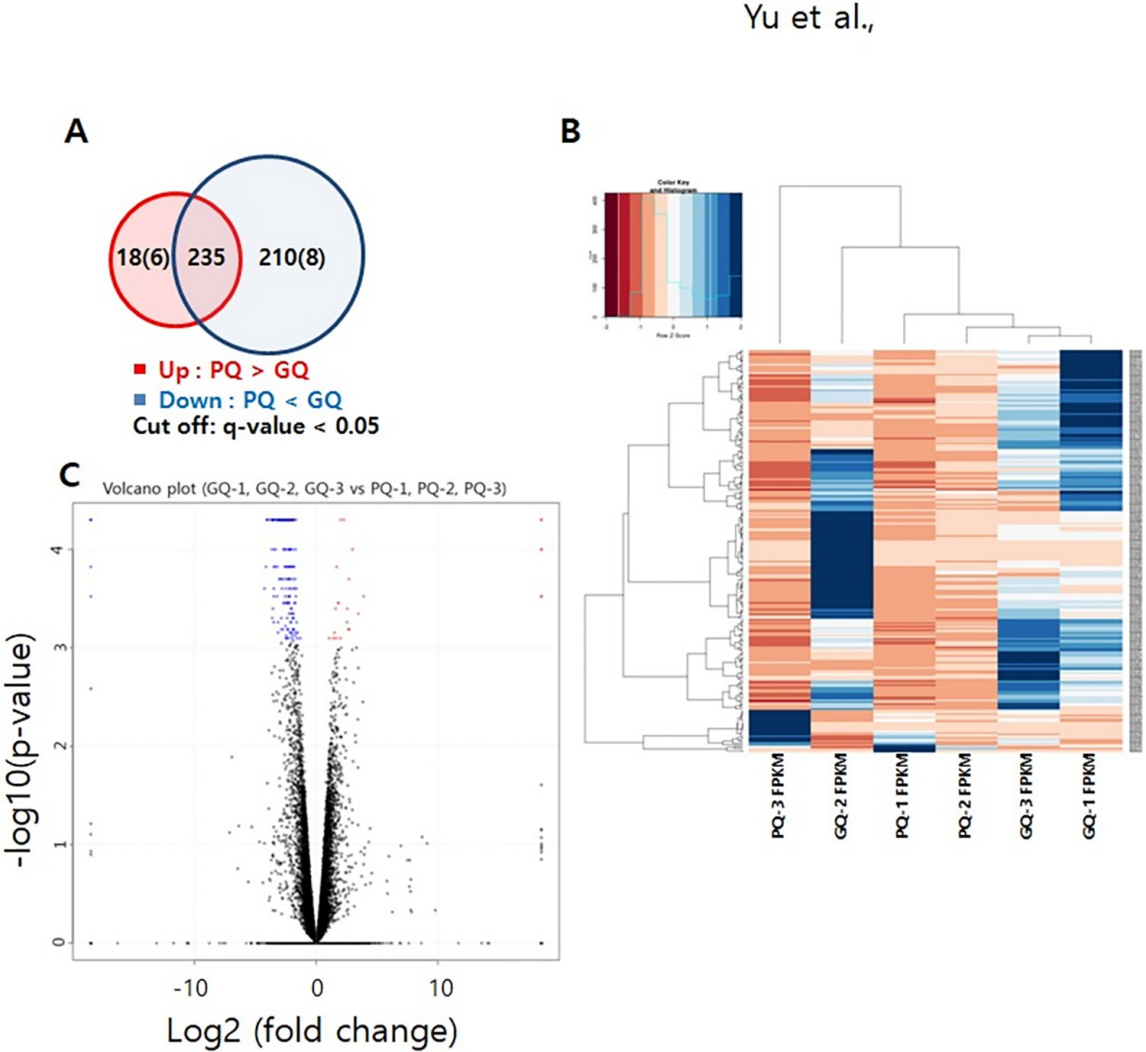
Hierarchical clustering and Differential Expression (DE) analysis of granulosa cells from good-quality (GQ) embryo transfer group compared with granulosa cells from poor-quality (PQ) embryo transfer group. (A) A Venn diagram depicting the distribution of significant differentially expressed genes (DEGs) (fold change > 2.5, q <0.05) in our study. Numbers represent the number of different genes showing upregulated or downregulated expression in PQ embryo samples compared with GQ embryo samples with red representing upregulated expression and blue indicating downregulated expression. Numbers in parenthesis represent the number of DEGs in only PQ or GQ group. (B) Heatmap of the DEGs between the GQ and PQ embryo groups. The color key from blue to red represents the relative gene expression level from low to high, respectively. (C) Volcano plot showing DEG analysis between GQ embryo and PQ embryo groups using DESeq2; 640 genes were differentially expressed (210 downregulated (FC < -2 and FDR < 0.05) and 25 upregulated (FC > 2 and FDR < 0.05). The upregulated genes are represented using red dots; downregulated genes are denoted using blue dots; and the black dots indicate genes with no significant changes.

Three GCs samples from each group were selected and used for RNA-seq analysis. Six hundred and forty genes were differentially expressed in our cohort. We analyzed DEGs between the two groups. Compared with that in the GQ embryo group, 18 genes were upregulated, whereas 208 genes were downregulated in the PQ embryo group (*p* < 0.05; FC of log2-transformed fragments per kilobase of transcript per million (FPKM) > 1) (Fig 2A). As shown in Fig 2B, we developed unsupervised hierarchical clustering of DEGs. A detailed list of 226 DEGs that were upregulated and downregulated in each group is presented in S2 Table.

### Gene ontology (GO) enrichment analysis: Differential expression revealed significant differences in gene expression regarding to the degree of embryo quality

**Fig 3:**
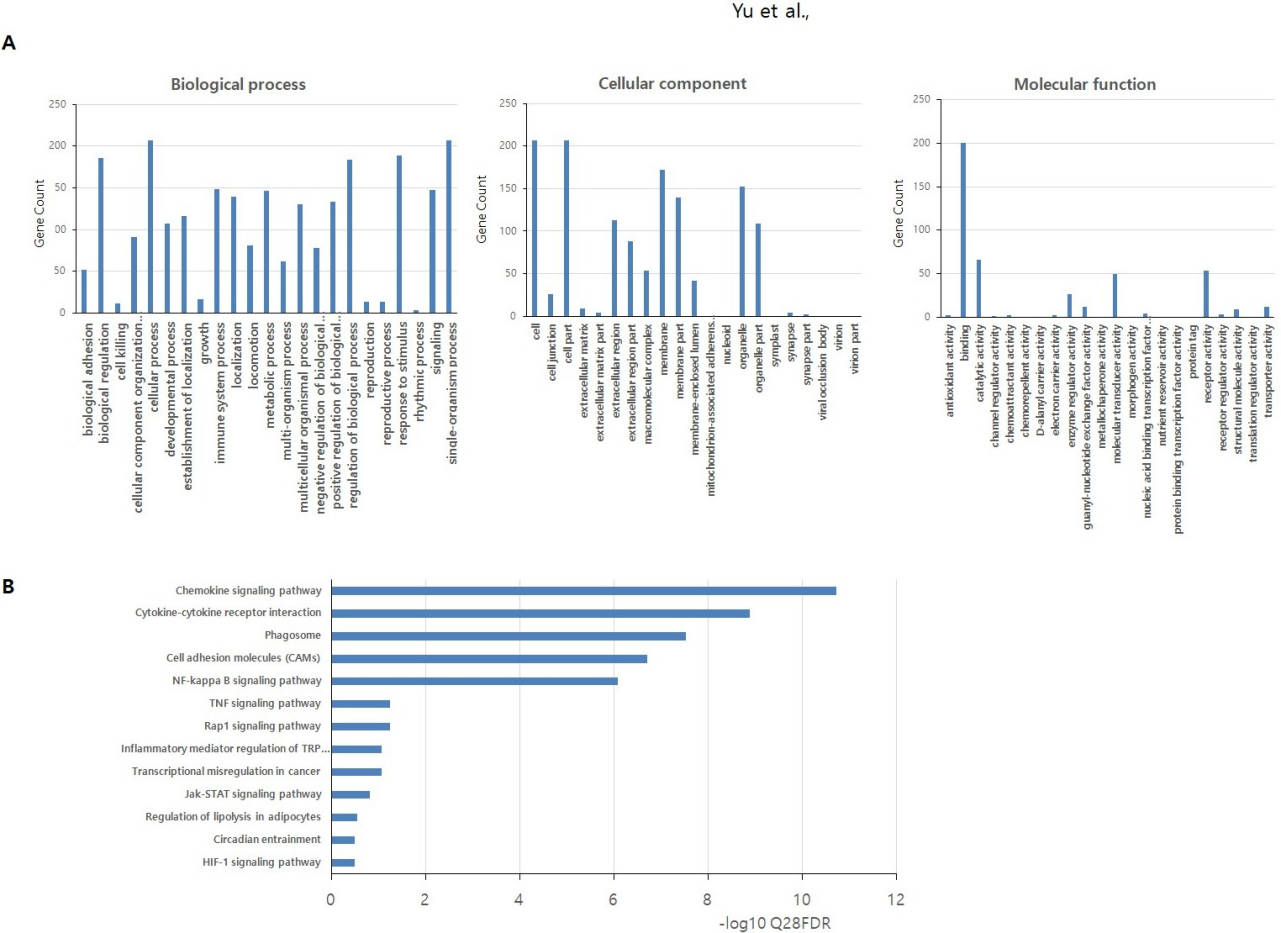
Histogram of the Gene Ontology(GO) analysis of the differentially expressed genes (DEGs). Genes were classified into three GO domains: biological process, cellular component, and molecular function. The left y-axis shows the gene count in each category. The solid columns indicate DEGs.

**Fig 4:**
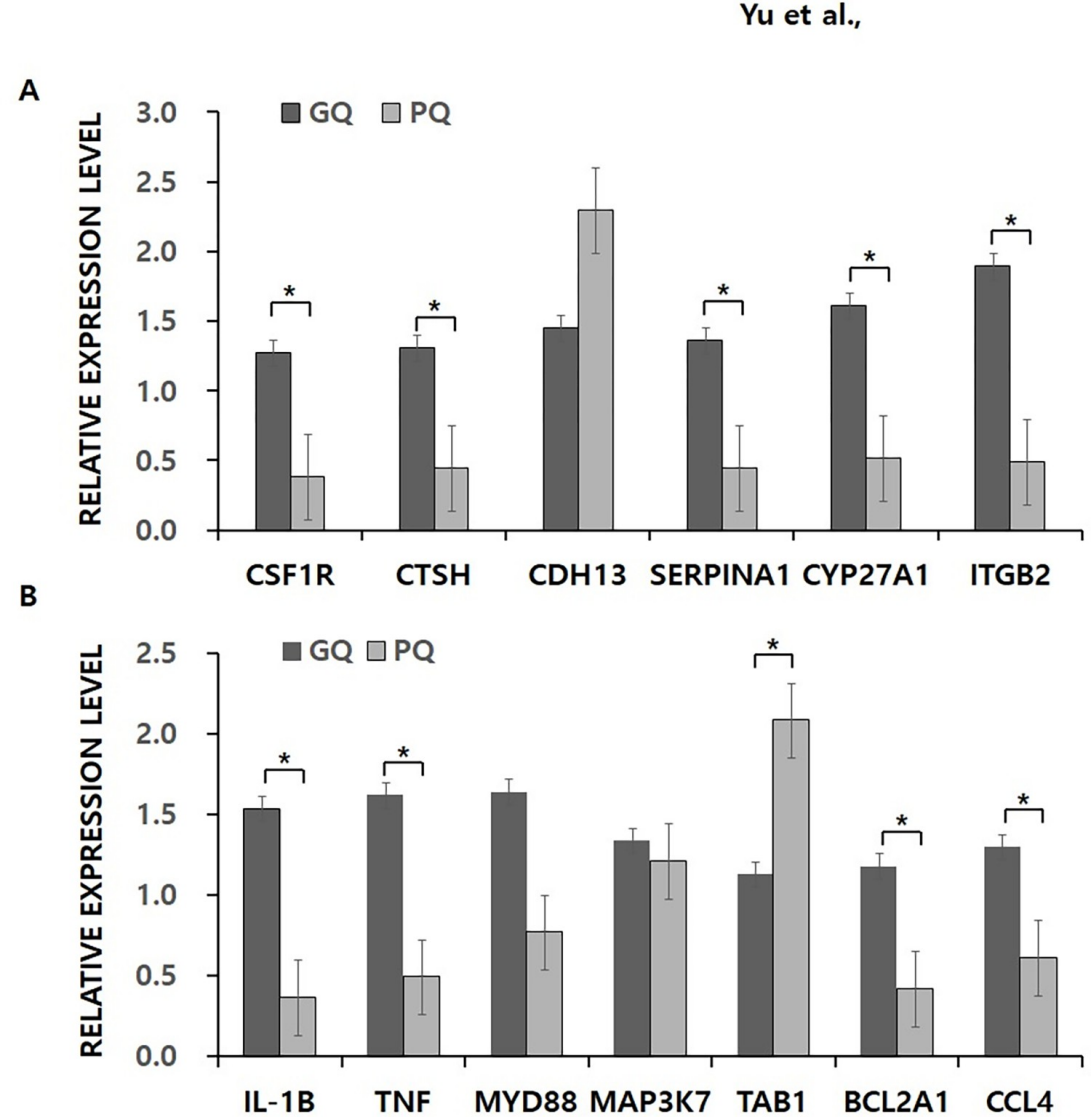
Validation of RNAseq results using RT-qPCR of 13 targets normalized to GAPDH in duplicate. Fold change was calculated using the 2^ΔΔCt^ method between the good-quality (GQ) and poor-quality (PQ) embryos. *p <0.05. (A) Six genes associated with embryo quality both in our and previous studies. (B) Seven genes randomly selected from the NF-kB pathway.

To further extend the molecular properties of the 226 DEGs, we performed a GO analysis of the up- or down-regulated groups (Fig 3). The DEGs were divided into three categories: biological processes, molecular functions, and cellular components. In the GO category biological process, DEGs were enriched in the response to stimulus, metabolic process, biological regulation, immune system process, binding and metabolic process, cellular process, single-organism process, cell and cell part, developmental process, cellular component organization or biogenesis, and the reproductive process. The top 20 genes displayed in the different embryo qualities are presented in Tables 2 and 3. KEGG pathway enrichment analysis showed that the DEGs in our study participated in 13 pathways (Fig 4). Several of these are related to reproduction, such as the chemokine signaling pathway, phagosome, cytokine-cytokine receptor interaction, cell adhesion molecules, and NF-kB signaling pathway.

**Table 2:** Top 20 up-regulated genes in the poor-quality embryo transfer group compared with the good-quality embryo transfer group.

GeneAcc	Gene name	Description	FC	p-value
ENSG00000175793	*SFN*	stratifin	3.90	0.0003
ENSG00000085465	*OVGP1*	oviductal glycoprotein 1	3.48	0.00045
ENSG00000140945	*CDH13*	cadherin 13	2.99	0.0001
ENSG00000231625	*SLC47A1P2*	SLC47A1 pseudogene 2	2.73	0.00065
ENSG00000112378	*PERP*	PERP, TP53 apoptosis effector	2.69	0.0002
ENSG00000015568	*RGPD5*	RANBP2-like and GRIP domain containing 5	2.65	0.00065
ENSG00000215252	*GOLGA8B*	golgin A8 family member B	2.56	0.0004
ENSG00000196436	*NPIPB15*	nuclear pore complex interacting protein family member B15	2.55	0.00055
ENSG00000121207	*LRAT*	lecithin retinol acyltransferase	2.28	5.00E-05
ENSG00000228486	*C2orf92*	chromosome 2 open reading frame 92	2.02	5.00E-05
ENSG00000163660	*CCNL1*	cyclin L1	2.00	0.0008
ENSG00000185864	*NPIPB4*	nuclear pore complex interacting protein family member B4	1.83	0.00035
ENSG00000153914	*SREK1*	splicing regulatory glutamic acid and lysine rich protein 1	1.83	0.00035
ENSG00000182389	*CACNB4*	calcium voltage-gated channel auxiliary subunit beta 4	1.69	0.00015
ENSG00000147854	*UHRF2*	ubiquitin like with PHD and ring finger domains 2	1.65	0.0008
ENSG00000111696	*NT5DC3*	5’-nucleotidase domain containing 3	1.61	0.0004
ENSG00000198408	*OGA*	O-GlcNAcase	1.51	0.0007
ENSG00000197745	*SCGB1D4*	secretoglobin family 1D member 4	inf	0.0001
ENSG00000161055	*SCGB3A1*	secretoglobin family 3A member 1	inf	0.0003

**Table 3:** Top 20 downregulated genes in the poor-quality embryo transfer group compared with the good-quality embryo transfer group.

GeneAcc	Gene name	Description	FC	p-value
ENSG00000142405	*NLRP12*	NLR family pyrin domain containing 12	-4.24	0.00025
ENSG00000211896	*IGHG1*	immunoglobulin heavy constant gamma 1 (G1m marker)	-4.12	0.00015
ENSG00000135929	*CYP27A1*	cytochrome P450 family 27 subfamily A member 1	-4.06	5.00E-05
ENSG00000104918	*RETN*	resistin	-4.04	5.00E-05
ENSG00000165457	*FOLR2*	folate receptor beta	-3.94	5.00E-05
ENSG00000132514	*CLEC10A*	C-type lectin domain containing 10A	-3.76	5.00E-05
ENSG00000072858	*SIDT1*	SID1 transmembrane family member 1	-3.64	0.0001
ENSG00000197766	*CFD*	complement factor D	-3.61	5.00E-05
ENSG00000134061	*CD180*	CD180 molecule	-3.61	0.0005
ENSG00000170458	*CD14*	CD14 molecule	-3.6	5.00E-05
ENSG00000158481	*CD1C*	CD1c molecule	-3.57	5.00E-05
ENSG00000102962	*CCL22*	C-C motif chemokine ligand 22	-3.54	5.00E-05
ENSG00000173369	*C1QB*	complement C1q B chain	-3.54	0.0004
ENSG00000155659	*VSIG4*	V-set and immunoglobulin domain containing 4	-3.53	5.00E-05
ENSG00000277632	*CCL3*	C-C motif chemokine ligand 3	-3.51	5.00E-05
ENSG00000072694	*FCGR2B*	Fc fragment of IgG receptor IIb	-3.48	0.0003
ENSG00000168546	*GFRA2*	GDNF family receptor alpha 2	-3.48	0.00055
ENSG00000113303	*BTNL8*	butyrophilin like 8	-3.47	0.00015
ENSG00000159189	*C1QC*	complement C1q C chain	-3.47	0.00025
ENSG00000142583	*SLC2A5*	solute carrier family 2 member 5	-3.45	0.0001

### Our findings enhanced the available literature exploring processes associated with embryo quality

When comparing our DEGs to those previously reported in the literature, Table 4 summarizes the findings of the detailed list of 68 genes that show genes positively associated with embryo quality. The GC gene biomarker studies have varied depending on the endpoints chosen, including ovarian development, follicular development, folliculogenesis, oocyte and embryo quality.

**Table 4:** Candidate genes associated with embryo quality.

Subject outcomes	Known Function	Sample	Method of detection	Gene	Previous study
Ovarian development	Signal transduction	Pooled GC	*Microarray*	*ACP5*, *ADCY7*, *AKAP9*, *ALPL*, *ARL5B*, *ATP2A1*, *COL16A1*, *DUSP2*, *GPR65*, *GPRC5C*, *HK3*, *ITGAL*, *KRT5*, *MAPK15*, *PIK3CG*, *PPF1A4*, *PRKCB*, *PTPN6*, *PTPRC*, *RAB7B*, *RGS19*, *TNFAIP2*, *TNFRSF10C*, *TYROBP*	[25–28]
Oocyte and embryo competence	Extra-cellular matrix proteases	Pooled GC	RNAseq, RT-qPCR	*ADAM8*, *ARHGDIB*, *ARHGEF33*, *CCL22*, *CD14*, *CD44*, *CYBA*, *CYP1B1*,*DHRS3*, *DOCK7*, *GPR137C*, *HIST1H4I*, *HLA-DPA1*, *IFI30*, *IL1B*, *LAPTM5*, *MMP9*, *PLA2G2D*, *PLD4*, *PLEK*, *PPBP*, *S100A8*, *TLR8*, *TMEM41B*, *TREM1*, *TRIM29*, *UCP2*,	[29–31]
Oocyte quality	Cytokine-cytokine receptor interaction, Signal transduction, Cytoskeleton organization, Chemokine signaling pathway	Pooled GC	Microarray, RT-qPCR	*AQP8*, *CXCL3*, *CXCR2*, *CYP27A1*, *ITBG2*, *OSM*, *PDE10A*, *TNFSF13*, *VAV1*,	[26, 32]
Embryo development	Apoptosis regulator	Embryo	RNAseq	*BCL2A1*, *SERPINA1*,	[33]
Folliculogenesis	Cytokine-cytokine interaction	Pooled GC	RNAseq	*CCL4*, *TNF*,	[34]
Follicle maturation	Extra-cellular matrix molecules	Pooled GC	RT-qPCR	*CDH13*, *DHCR7*	[35, 36]
Follicular survival	Cellular protease	Pooled GC	RT-qPCR	*CTSH*	[37]
Follicular development	Growth factor	Individual GC	RT-qPCR	*CSF1R*	[38]

GC, granulosa cells; RT-qPCR, reverse transcription- quantitative polymerase chain reaction; RNA seq, ribonucleic acid sequencing

### Validation of DEGs using RT-qPCR analysis

To examine the reliability of the RNA-seq data, we selected 13 genes to verify their expression in GC using RT-qPCR (Fig 4). When comparing our DEGs to those reported in previous studies, we found six genes that were related to embryo quality both in our study and previous published literature, including colony stimulating factor receptor 1 (CSFR1) [38], cathepsin H (CTSH) [37], cadherin 13 (CDH13) [35], serpin family A member 1 (SERPINA1) [39], cytochrome P450 family 27 subfamily A member 1 (CYP27A1) [40], and integrin subunit beta 2 (ITGB2) [16]. Among the significantly enriched pathways, we randomly selected seven genes from the NF-kB pathway: four genes that have refuting findings between different studies (interleukin 1 beta (IL1β), tumor necrosis factor (TNF), BCL2 related protein A1 (BCL2A1), C-C motif chemokine ligand 4 (CCL4)), and three genes that did not support an association with pregnancy or embryo quality (MYD88 innate immune signal transduction adaptor(MYD88), mitogen-activated protein kinase kinase kinase 7(MAP3K7), and TGF-beta activated kinase 1 binding protein 1(TAB1)).

Thirteen candidate genes, comprising eleven downregulated genes in GCs from only PQ embryo groups, CSF1R, CTSH, IL1β, TNF, BCL2A1, CCL4, SERPINA1, CYP27A1, ITGB2, MYD88, MAP3K7, and two upregulated genes in GC from PQ embryo groups, TAB1, and CDH13, were selected and analyzed using RT-qPCR. The RT-qPCR results were consistent with the RNA-seq data, which means that the RNA-seq results were dependable and that it could be used to perform accurate differential expression analysis of mRNA.

## Discussion

In the present study, we analyzed GC expression of CSF1R, CTSH, IL1β, TNF, BCL2A1, CCL4, SERPINA1, CYP27A1, ITGB2 from GCs collected during IVF from oocytes that developed into GQ embryos. Selected genes were shown to be well differentiated between immature MI and mature MII oocytes. In recent years, many studies have been performed to analyze GC expression in association with various endpoints: oocyte quality, embryo development and pregnancy (Table 4). Since GC is an easily accessible material that is normally discarded during the IVF cycle, it represents a good biological material for research and diagnostic purposes.

Several signaling pathways are important in mediating or modifying the proliferative response of GCs to FSH stimulation, the primary mediator of GC proliferation during folliculogenesis [41]. Our RNA-Seq results showed that some differential pathways might verify oocyte development, such as the chemokine signaling pathway, cytokine-cytokine receptor interaction, phagosome, cell adhesion molecules, and NF-kB signaling pathway. Thus, we could infer that the PQ embryos were mainly related to the expression levels of cytokine reaction, apoptosis, and the adherent junction pathway.

In our study, the differential expression of transcripts in the NF-kB signaling pathway was related to embryo quality (Fig 4B). This pathway participate in apoptosis and cell growth, as well as in immune, inflammatory and acute phase responses [42]. The downregulation of immune and inflammation-related genes in the PQ embryo group was in agreement with the hypothesis that ovulatory process is an acute inflammatory reaction [43]. Consistent with previous studies [44, 45], various ovulation-associated factors such as PDE2A (3′5’-cyclic nucleotide phosphodiesterase 2A), RGS1 and RGS16 (a regulator of G-protein signaling 1 and 16), ADAMTS1 (a disintegrin-like and metalloprotease with thrombospondin type 1 motif, 1), and PTGS1 (prostaglandin-endoperoxide synthetase 1), were all upregulated in GCs from the PQ embryo group.

CSF-1R is activated in a similar way by homodimeric growth factors colony-stimulating factor-1 (CSF-1) and interleukin (IL)-34 [46]. CSF-1 may be essential in regulating the response of GCs to gonadotropin and may promote in the early embryonic development [38]. The biological activity of CSF-1 depends on its binding to CSF-1R in target cells [47].

CTSH expression is increased in human GCs during follicular maturation in vivo [37]. Our study demonstrated that the expression of CTSH was higher in GCs from GQ embryos than that from PQ embryos with statistical significance, suggesting embryo development failure was caused by poor follicular maturation and GC dysfunction.

SERPINA1 is the gene for an acute-phase protein that increases in response to inflammation [48]. This gene, implicated in the inflammatory response, reduces the levels of proinflammatory mediators, and increases anti-inflammatory cytokines [49]. In our study, increased SERPINA1 expression levels were observed in GQ embryo. Overexpression of SERPINA1 in GCs from the GQ embryo group indicates decreased proinflammatory cytokine levels, which leads to increased developmental competence. This finding suggests that an impaired follicular fluid microenvironment characterized by elevated pro-inflammatory cytokines may cause poor quality of oocyte.

Changes in the cellular structure of the corpus luteum (CL) on natural luteolysis may contribute to elevated concentrations of CYP27A1 mRNA. For example, macrophages invade the CL during luteolysis and express CYP27A1 [50]. In addition, increased CYP27A1 protein concentrations in mitochondria cause elevated conversion of cholesterol into 27OH, which decreases progesterone secretion in human luteinized GC [40].

ITGB2 is an integrin subunit that is a pivotal mediator of uterine receptivity and embryonic development [51]. Overexpression of this gene is associated with GCs of healthy follicles and decreased with atresia [52].

Reorganization of cellular composition for ovulation includes follicular wall degradation, and oocyte expulsion during the advanced stages of follicular development [53]. IL1β, TNF-α, and CCL4 are pro-inflammatory cytokines that act locally on ovarian follicular cells and are involved in the ovulation process [54]. During the periovulatory phase, ovarian macrophages collect in the ovary and secret these pro-inflammatory cytokines [55–57]. They regulate the secretion of steroid hormones for follicle growth and are important for ovulation process and the development and regression of the CL [58, 59]. For instance, IL-1β has been shown to induce ovulation by promoting follicular rupture [60]. In the present study, IL1β, TNF-α, and CCL4 were significantly upregulated in GCs from oocytes that yielded GQ embryos. This finding suggests that these cytokines may modulate oocyte quality and embryo development.

TAB1 may represents the initiation of inflammatory functions and may serve as a connector of the IL-1 induced signaling pathway leading to activation of c-Jun N-terminal kinase (JNK) and NF-κB signaling pathways [61, 62]. B-cell lymphoma-2 (BCL2) family proteins are pivotal regulators of apoptosis [63]. The ovaries of *Bcl2* knockout mice were revealed to have fewer primordial follicles [64]. However, this loss may be due to GC apoptosis, which indirectly affects follicle development. BCL2 family members, such as *BCL2A1*, are expressed during embryonic genome activation and maintained until the blastocyst stage [33], suggesting that these are required throughout early embryonic development.

The strength of our study is that it was prospective and sampled a large group of patients who were followed up clinically. We also performed sorting and enrichment analysis of RNA-sequencing data, the most unbiased approach that can be used to explore transcriptomic signatures [65]. Furthermore, by incorporating information from DEGs, pathway analysis, and related studies, the present study provides a list of candidate genes with functions and expression levels closely related to embryo quality in IVF. In this study, RNA-sequencing data of whole GCs collection revealed a difference between the group that produced good-quality embryos and the group that produced only poor-quality embryos. This difference could be used for therapeutic purposes to improve embryo quality in the future.

The present study, however, has some limitations. First, GCs obtained from the follicular fluid were GCs in all follicles and cannot accurately reflect the quality of single oocyte. In clinical practice, it may be more difficult to collect GCs from a single follicle than cumulus cells. Second, we reported expression levels of the genes, but did not analyze expression ot corresponding proteins and the role of post-translational modifications.

## Conclusions

We found that the expression profiles of specific genes in GCs was associated with embryo quality during IVF and suggested the need to develop treatment strategies that can compensate for the poor quality due to the deficiency of specific genes during follicular development. Furthermore, we reported the published data that support or confute granulosa expressed genes as biomarkers for identifying oocyte or embryo quality. This study improves our understanding of reproductive function of GCs, which could be helpful for more targeted studies aiming to improve oocyte and embryo competence in the future.

## Supporting information

S1 Table Primer sequences used for real-time qPCR. (DOCX)

S2 Table A detailed list of 226 DEGs that were upregulated and downregulated in good-quality and poor-quality embryo group. (XLSX)
