# Supplementary material for: RNA sequencing-based transcriptome analysis of granulosa cells from follicular fluid: Genes involved in embryo quality during in vitro fertilization and embryo transfer
Source: PLoS One. 2023 Mar 1;18(3):e0280495. doi: 10.1371/journal.pone.0280495 (PMC9977003; doi:10.1371/journal.pone.0280495)
Supplement: S1 Table — (DOCX) [file pone.0280495.s001.docx]

(Supplemental) table S2. Primer sequences used in RT-qPCR

|  | |  |  |
| --- | --- | --- | --- |
| **Gene** | **Sense primer** | **Antisense primer** | **Amplicon (bp)** |
| CSF1R | CTGCCCAGATCGTGTGCTC | AGGTTGAGGGTCAGGACTTTTT | 139 |
| CTSH | AAACGCCCACAACAATGGGA | TGGTGGCTGAGCAATTCTGAG | 122 |
| CDH13 | AGTGTTCCATATCAATCAGCCAG | CGAGACCTCATAGCGTAGCTT | 106 |
| SERPINA1 | GATCAACGATTACGTGGAGAAGG | CCTAAACGCTTCATCATAGGCA | 207 |
| CYP27A1 | CAGCACGACCTGACCTATGG | TGGTCCAGTCGAGTCATAAAGT | 158 |
| ITGB2 | TGCGTCCTCTCTCAGGAGTG | GGTCCATGATGTCGTCAGCC | 187 |
| IL-1B | ATGATGGCTTATTACAGTGGCAA | GTCGGAGATTCGTAGCTGGA | 132 |
| TNF | TGCTTGTTCCTCAGCCTCTT | TGAGGTACAGGCCCTCTGAT | 313 |
| MYD88 | CGGAGGAGATGGACTTTGAG | TCATCTCCTGCACAAACTGG | 384 |
| MAP3K7 | ACAGTGTTCCCAAGGAGTGG | CAGGCTCTCAATGGGCTTAG | 382 |
| TAB1 | CCAGCTGCCTCCTCAGTATC | AGGTCAATGTCCGTGTAGCC | 345 |
| BCL2A1 | CCGTAGACACTGCCAGAACA | ACATGGGGACAAAATTTCCA | 487 |
| CCL4 | GAGTTCTGCAGCCTCACCTC | GCTTGCTTCTTTTGGTTTGG | 261 |
